# Beyond Average Sex Differences: A Bayesian Analysis of Extreme and Discordant Physical Activity–Dietary Behavioral Profiles in University Students

**DOI:** 10.3390/nu18203379

**Published:** 2026-10-15

**Authors:** Jarosław Domaradzki

**Affiliations:** Department of Biological Principles of Physical Activity, Wroclaw University of Health and Sport Sciences, 51-612 Wrocław, Poland; jaroslaw.domaradzki@awf.wroc.pl

**Keywords:** physical activity, dietary behaviors, behavioral discordance, Bayesian mixture modelling, university students

## Abstract

**Background/Objectives**: Physical activity (PA) and dietary behaviors are commonly examined separately or through average associations, potentially obscuring individual heterogeneity and behavioral discordance. This study aimed to identify extreme and recurring PA–dietary behavioral profiles and examine sex-related differences. **Methods**: This cross-sectional study included 418 university students (199 men and 219 women). Physical activity was assessed using the International Physical Activity Questionnaire (IPAQ-LF), whereas dietary behaviors were evaluated using the Questionnaire of Eating Behaviors (QEB) and expressed as the pro-Healthy Diet Index (pHDI-8) and non-Healthy Diet Index (nHDI-8). Robust Mahalanobis distance (RMD) was used to identify extreme multivariate configurations. Bayesian finite-mixture modelling was subsequently used to explore recurring behavioral profiles, and concordant and discordant PA–dietary configurations were examined by sex. **Results**: RMD identified 49 participants (11.7%) with extreme multivariate configurations; these configurations were significantly more frequent among men than women (15.1% vs. 8.7%, *p* = 0.042). Bayesian modelling yielded a converged, exploratory two-profile solution differentiated primarily by dietary behaviors. The more favorable dietary profile included 37.1% of participants and was more frequent among women (54.8% vs. 17.6%; OR = 5.68, 95% CI: 3.62–8.92). Discordant PA–dietary configurations occurred in 49.5% of the sample and were more frequent among women (57.1% vs. 41.2%; OR = 1.90, 95% CI: 1.29–2.80). Women predominantly exhibited lower PA and more favorable dietary behaviors, whereas men more frequently exhibited higher PA and less favorable dietary behaviors. Discordance was not associated with multivariate extremeness (*p* = 0.823). **Conclusions**: Joint assessment of PA and dietary behaviors revealed heterogeneity beyond average sex differences. Discordant PA–dietary configurations differed by sex, although their distribution was sensitive to reference cutoffs. Discordance and multivariate extremeness represented distinct aspects of behavioral heterogeneity. These findings support person-centered assessment of lifestyle behaviors and may inform more targeted health-promotion strategies.

## 1. Introduction

Physical activity and dietary behaviors are important components of lifestyle and may be related to health outcomes [1]. Their combination may also be relevant, as individuals exhibiting healthier patterns of both physical activity and diet tend to show more favorable health profiles than those with less healthy behavioral combinations [2]. However, physical activity and dietary behaviors do not necessarily cluster in a consistently healthy or unhealthy manner. High levels of physical activity may coexist with frequent unhealthy dietary behaviors [3], indicating that being physically active does not necessarily imply adherence to a healthy diet. Previous studies have also shown that the relationship between physical activity and diet may differ depending on the dietary behavior considered. More physically active individuals have been reported to consume greater amounts of health-promoting foods and nutrients, including fruits and vegetables, fiber, calcium, folate, and vitamins A, C, and E, whereas associations with saturated fat, trans fat, and dietary cholesterol followed different patterns [4]. Importantly, healthy and unhealthy dietary behaviors appear to have partly distinct correlates rather than representing opposite ends of a single behavioral continuum [5]. Consequently, considering them as separate dimensions may provide a more informative framework for examining how dietary behaviors coexist with physical activity. At the most general level, health behaviors form two broad categories or dimensions: preventive behavior and risk-taking behavior [6].

Studies examining physical activity and diet commonly focus on individual behaviors or their average associations with health outcomes [7]. Although such approaches are useful for describing overall tendencies, they may obscure how different behaviors coexist within individuals. For example, high physical activity may coexist with frequent unhealthy and infrequent healthy dietary behaviors, a configuration that may not be evident from group-level means or correlations [8]. Indeed, a study of 700 adults found no average within-person association between physical activity and fruit and vegetable intake despite substantial interindividual variability in this relationship; however, unhealthy dietary behaviors were not considered. These findings suggest that average associations may conceal heterogeneous, and potentially discordant, combinations of physical activity and dietary behaviors [9].

Identifying a person with such an extreme combination does not establish whether a similar configuration occurs elsewhere in the population at less extreme levels [10]. The key question is whether discordant combinations are isolated observations or part of a recurring behavioral pattern. This distinction requires examining both unusual individual configurations and profiles shared by multiple participants.

Previous studies have partly addressed the coexistence of physical activity and dietary behaviors by examining their associations and combined relationships with health outcomes [2,7]. Person-centered approaches have further identified distinct combinations of dietary and physical activity behaviors [8], while a systematic review has highlighted the heterogeneity of behavioral clusters and their associations with physical and mental health outcomes [1]. However, these approaches have not specifically distinguished between multivariate extremeness and behavioral discordance, nor established whether discordant configurations are confined to extreme observations or also occur more broadly within a population. Addressing this distinction may improve the characterization of individual behavioral heterogeneity and inform more differentiated health-promotion strategies.

Recent methodological developments have expanded the possibilities for examining heterogeneity in multidimensional data, including robust distance-based approaches for identifying atypical observations [11] and Bayesian mixture models for investigating latent population heterogeneity [12]. These approaches offer complementary perspectives on individual differences, distinguishing unusual multivariate configurations from recurring patterns within a population. Importantly, multivariate extremeness does not necessarily imply behavioral discordance, which requires consideration of the direction and combination of physical activity and dietary behaviors.

The aim of this study was to determine whether discordant combinations of physical activity and dietary behaviors are limited to multivariate extreme cases or represent a broader feature of behavioral heterogeneity within the population. Specifically, the study examined multivariate extreme configurations, recurring behavioral profiles, the prevalence and overlap of discordance and extremeness, and sex-related differences in these behavioral patterns. We hypothesized that discordant physical activity–dietary configurations would extend beyond multivariate extreme cases and would also occur among participants with less extreme behavioral values.

## 2. Materials and Methods

### 2.1. Study Design

A cross-sectional design was used in the present study, which forms part of a larger research programme investigating lifestyle-related behaviours and their health correlates among physically active young adults. The data were derived from two student cohorts recruited at the Wroclaw University of Health and Sport Sciences during 2022–2023. Both cohorts were examined according to the same standardized research protocol, allowing their data to be combined for the present analyses. Participants with complete information on dietary behaviours, physical activity, anthropometric measures, and body composition were included in the analytical dataset. Dietary behaviours were evaluated using the Questionnaire of Eating Behaviour (QEB), while physical activity was assessed with the long form of the International Physical Activity Questionnaire (IPAQ-LF).

### 2.2. Ethics

The research protocol received approval from the Senate Research Ethics Committee of the Wroclaw University of Health and Sport Sciences (No. 13/2022). Before data collection, participants received information regarding the purpose and procedures of the study and provided electronic informed consent to participate.

### 2.3. Sample Size

The minimum sample size was determined using the standard formula for estimating a population proportion at a 95% confidence level:

$$n=(\frac{1.96}{\delta }{)}^{2}\times p\left(1-p\right)$$

where *δ* represents the margin of error and *p* is the expected proportion.

Using the most conservative assumption of *p* = 0.50 and a margin of error of 5%, the minimum required sample was calculated as 384 participants. To account for potentially incomplete records, the recruitment target was increased to approximately 460 individuals. Following data completeness screening, 418 participants with all variables required for the present analyses were included in the final analytical sample.

### 2.4. Participants

Participants were full-time university students aged 18–25 years who attended classes in person at a university specializing in physical education and sport sciences. No minimum physical activity threshold was applied as an eligibility criterion. Physical activity levels varied among participants and were assessed as one of the principal study variables. Exclusion criteria comprised participation in university-regulated elite sport programmes, chronic metabolic or psychiatric conditions, night-shift work, and implausible questionnaire responses.

**Figure 1:**
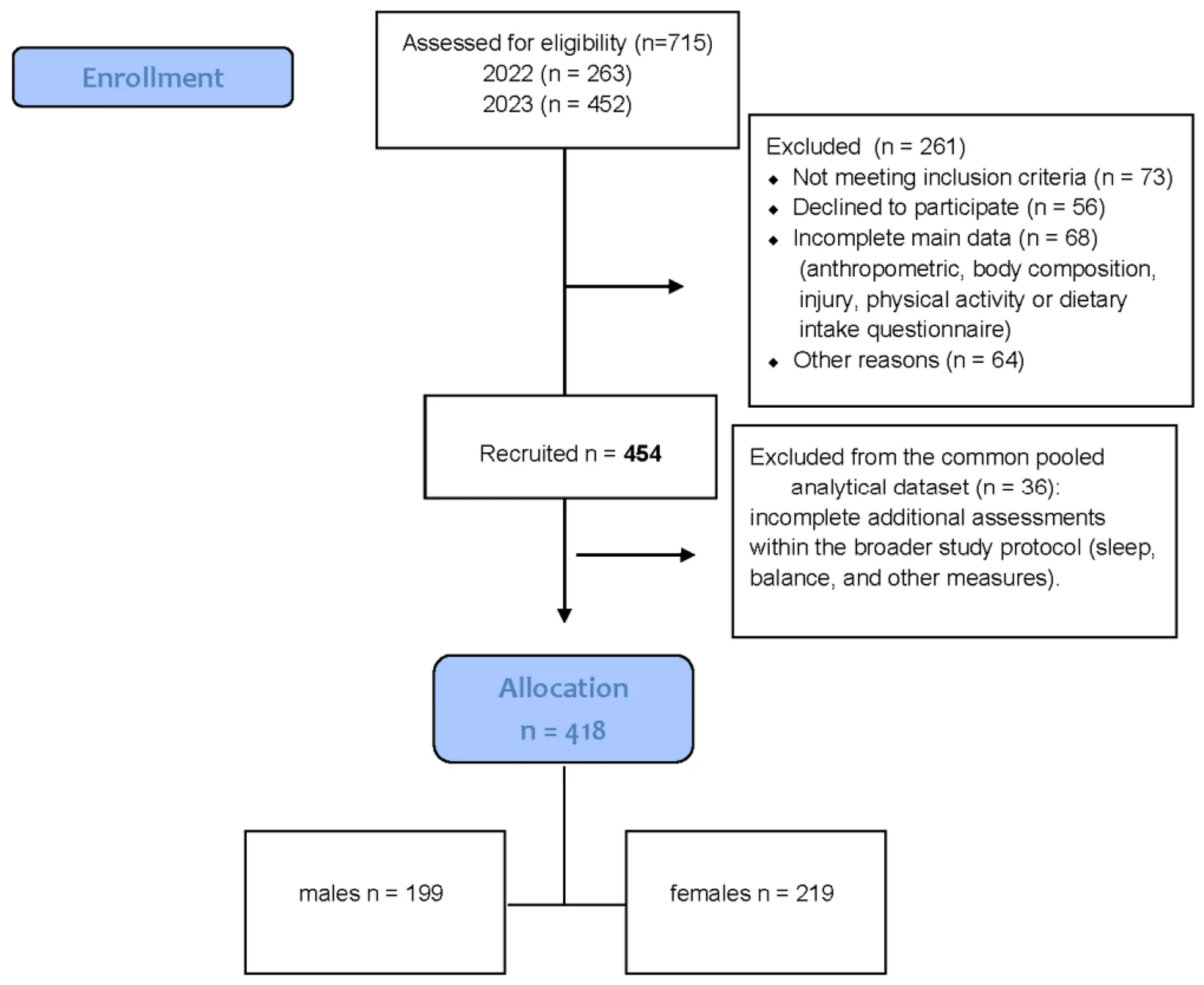
Flow diagram of the progress through all phases of data collection.

Across the two cohorts, 454 students were initially recruited. After applying the eligibility criteria and screening the data for completeness and quality, 418 participants were retained for analysis, including 199 men (age: 20.73 ± 0.85 years) and 219 women (age: 20.56 ± 0.74 years). The 36 excluded participants had incomplete additional assessments within the broader research protocol, whereas complete data were available for all variables analyzed in the present study. No imputation was performed for these variables (Section 2.7). The participant selection process is presented in Figure 1.

Before combining the cohorts, their comparability was examined with respect to age, sex, BMI, dietary behaviours, and physical activity. None of these characteristics differed significantly between cohorts (all *p* > 0.05), providing support for their inclusion in a pooled analytical sample.

### 2.5. Anthropometric and Body Composition Measurements

Anthropometric and body composition assessments were conducted under standardized laboratory conditions at the Biokinetics Research Laboratory. Body height was measured to the nearest 0.1 cm using a GPM anthropometer (GPM Instruments GmbH, Switzerland), while body mass, body fat mass (BF), body fat percentage (BF%), and fat-free mass (FFM) were assessed by bioelectrical impedance analysis using an InBody230 analyzer (InBody Co., Seoul, South Korea). Body mass index (BMI) was calculated as body mass divided by height squared (kg/m^2^).

### 2.6. Questionnaire Measurements

#### 2.6.1. Physical Activity—International Physical Activity Questionnaire (IPAQ)

Physical activity was evaluated using the validated Polish version of the International Physical Activity Questionnaire—Long Form (IPAQ-LF) [13]. Questionnaire responses were processed according to the standard IPAQ scoring procedure and expressed as metabolic equivalent minutes per week (MET-min/week). Total physical activity was used as the physical activity dimension in the subsequent behavioral profile analyses.

#### 2.6.2. Dietary Intake Questionnaire—Questionnaire Eating Behaviours (QEB)

Dietary behaviours were evaluated using the validated 16-item Questionnaire of Eating Behaviours (QEB) [14,15]. Reported consumption frequencies were converted into daily frequencies (0, 0.06, 0.14, 0.5, 1, or 2 times/day, depending on the response category) and aggregated into two eight-item indices: the pro-Healthy Diet Index (pHDI-8) and the non-Healthy Diet Index (nHDI-8). The pHDI-8 comprised wholemeal bread, milk, fermented milk beverages, cottage cheese, fish, legumes, fruit, and vegetables, whereas the nHDI-8 comprised fast food, fried foods, yellow cheese, sweets and confectionery, canned foods, sugar-sweetened carbonated beverages, energy drinks, and alcoholic beverages. Each index was calculated by summing the daily consumption frequencies of its eight components and converting the result to a 0–100-point scale using the formula: index score = (100/16) × sum of daily consumption frequencies. These indices represent the frequency of healthy and unhealthy dietary behaviours, respectively, and were used as the two dietary dimensions of the behavioral profile analyses.

For descriptive presentation, the dietary indices were Yeo–Johnson transformed, whereas subsequent multivariate analyses used their standardized z-scores, as detailed in Section 2.8.

### 2.7. Analytical Sample Selection and Missing Data Handling

The present study forms part of a series of exploratory investigations based on a common pooled dataset collected across two university student cohorts. To maintain consistency and comparability across related analyses addressing different behavioral, psychological, and functional outcomes, the same analytical sample of 418 participants was retained. Of the 454 recruited participants, 36 were excluded from the common analytical dataset because of incomplete additional assessments within the broader research protocol, including sleep-related questionnaires, balance measurements, and other measures not examined in the present study.

Missing-data patterns were evaluated within the broader research protocol, including consideration of the Missing Completely at Random (MCAR) assumption [16]. All 418 participants retained for the present study had complete data for the variables included in the analyses. No missing-data imputation was performed for these variables.

### 2.8. Statistics

Descriptive statistics were calculated for the total sample and separately for women and men. Age, body height, body mass, BMI, body fat mass (BF), body fat percentage (BF%), and fat-free mass (FFM) were used to characterize the study sample, whereas total physical activity (PA), the pro-Healthy Diet Index (pHDI-8), and the non-Healthy Diet Index (nHDI-8) constituted the three behavioral dimensions used in the multivariate analyses. Between-sex comparisons were performed using appropriate tests according to variable distribution.

Prior to multivariate analyses, PA, pHDI-8, and nHDI-8 were transformed using the Yeo–Johnson transformation and subsequently standardized to z-scores. In Table 1, PA is presented on its original scale (MET-min/week), whereas pHDI-8 and nHDI-8 are presented as Yeo–Johnson-transformed but non-standardized scores.

First, multivariate atypicality within the joint PA–pHDI-8–nHDI-8 space was evaluated using robust Mahalanobis distance (RMD). Robust estimates of multivariate location and covariance were obtained using the minimum covariance determinant approach. Extreme multivariate configurations were defined using the 97.5th percentile of the chi-square distribution with three degrees of freedom (RMD^2^ > 9.35; RMD > 3.06). The prevalence of RMD-defined extreme configurations was compared between women and men.

Second, Bayesian finite-mixture modelling was applied to identify recurring profiles within the joint distribution of standardized PA, pHDI-8, and nHDI-8. Models containing one to five components were estimated. Mixture proportions were assigned a symmetric Dirichlet (1) prior, profile-specific means normal priors (N [0, 1.5]), standard deviations half-normal priors (N [0, 1], constrained to positive values), and profile-specific correlation matrices an LKJ prior with shape parameter 2. To reduce label switching, profile-specific means for pHDI-8 were constrained to an ordered structure. Models were estimated using four Markov chain Monte Carlo chains, each with 2000 iterations, including 1000 warm-up iterations, with adapt_delta = 0.95 and maximum tree depth = 12. Convergence and sampling quality were evaluated using R-hat, effective sample size (ESS), divergent transitions, and maximum-tree-depth transitions. Models with inadequate convergence were excluded from posterior interpretation and predictive comparison. A targeted sensitivity analysis of the three-component model used informed initialization, 3000 iterations per chain (1500 warm-up), adapt_delta = 0.99, and maximum tree depth = 15 (Tables S1 and S2). For converged candidate models, predictive performance was compared using Pareto-smoothed importance-sampling leave-one-out cross-validation (PSIS-LOO), including expected log predictive density (ELPD-LOO), leave-one-out information criterion (LOOIC), and Pareto k diagnostics (Supplementary Table S3). Individual profile membership was determined from posterior class-membership probabilities, and classification certainty was quantified using the maximum posterior membership probability. Posterior profile-specific means and corresponding 95% credible intervals (CrIs) were used to characterize each profile. Posterior predictive checks compared observed and model-replicated distributions and summary statistics (Figure S1 and Table S7).

Third, concordant and discordant PA–dietary configurations were examined independently of Bayesian profile membership. Overall dietary favorability was calculated as the difference between standardized pHDI-8 and nHDI-8 scores (pHDI-8_z—nHDI-8_z) and subsequently standardized, with higher values indicating more favorable dietary behavior. Participants were classified according to whether standardized PA and dietary favorability were above or below their pooled-sample means. This yielded four configurations: concordant favorable (higher PA/more favorable diet), concordant unfavorable (lower PA/less favorable diet), discordant higher PA/less favorable diet, and discordant lower PA/more favorable diet. The latter two configurations were combined to represent overall behavioral discordance. To assess the sensitivity of this classification to sex differences in the underlying behavioral distributions, the four configurations were additionally defined using sex-specific mean cutoffs for standardized PA and dietary favorability. The resulting distributions and between-sex differences were compared with those obtained using pooled-sample cutoffs (Supplementary Table S4). Sex differences in Bayesian profile membership, overall discordance, and the distribution of the four behavioral configurations were examined using contingency-table analyses and binary logistic or multinomial logistic regression, as appropriate. Finally, associations between behavioral discordance and RMD-defined multivariate extremeness, as well as differences in the prevalence of RMD-defined extreme cases across the four behavioral configurations, were examined.

Statistical significance for frequentist analyses was set at *p* < 0.05, with effect estimates reported as odds ratios (ORs) and 95% confidence intervals (CIs), where applicable. Bayesian inference was based on posterior distributions and 95% CrIs, with model adequacy evaluated using convergence diagnostics and out-of-sample predictive performance.

### 2.9. AI Transparency Statement

Generative artificial intelligence (AI) tools were used during manuscript preparation in accordance with COPE guidance and MDPI recommendations concerning transparency in AI-assisted scientific writing. Chat Academia (v.1.0, 2025) and Elicit (v.2.0, 2025) were employed to support literature exploration and the development and refinement of research questions, while SciSpace (2025) assisted with literature organization and review. Julius AI (2025) and ChatGPT (OpenAI, GPT-4.1 and GPT-5.6, 2025–2026) were used for methodological consultation, editorial and language support, and selected technical tasks, including verification and troubleshooting of R code.

AI-assisted tools were not used for data collection. All AI-generated outputs, including literature-related suggestions, methodological recommendations, text revisions, and code fragments, were critically evaluated and independently verified by the authors before use. All final decisions regarding study methodology, statistical analyses, interpretation of findings, and manuscript content remained the responsibility of the authors, who take full responsibility for the accuracy, integrity, and reproducibility of the reported work.

## 3. Results

### 3.1. Participant Characteristics

The general characteristics of the study sample are presented in Table 1. Significant sex differences were observed across anthropometric characteristics, body composition, physical activity, and dietary behaviours. Males were taller and heavier and had higher BMI and physical activity levels than females (all *p* < 0.001). Body composition also differed between sexes: males had higher fat-free mass, whereas females had higher body fat percentage and absolute body fat mass. Dietary behaviours showed clear sex-related differences, with females reporting higher pro-Healthy Diet Index (pHDI-8) scores and males higher non-Healthy Diet Index (nHDI-8) scores (both *p* < 0.001). These group-level differences provide the descriptive context for the subsequent analyses focused on individual heterogeneity and extreme or discordant physical activity–dietary behavioural profiles beyond average sex differences.

### 3.2. Identification of Multivariate Extreme Cases in Physical Activity and Dietary Behaviors Using Robust Mahalanobis Distance

Robust Mahalanobis distance (RMD) analysis identified 49 participants (11.7%) as exhibiting atypical multivariate configurations of physical activity and dietary behaviors, based on the predefined cutoff of RMD > 3.06 (RMD^2^ > 9.35). The prevalence of extreme profiles was higher among men than women (15.1% vs. 8.7%, respectively), with a statistically significant between-sex difference (Pearson’s χ^2^(1) = 4.13, *p* = 0.042). The median RMD was 1.65 in the total sample and was higher in men than women (1.79 vs. 1.53), with maximum values of 5.70 and 5.46, respectively (Table 2).

Inspection of the standardized behavioral dimensions indicated that multivariate extremeness was not attributable to a single behavioral component. Rather, extreme cases represented heterogeneous combinations of physical activity, pHDI-8, and nHDI-8. For example, high RMD values occurred for profiles combining relatively high physical activity with low pHDI-8 and nHDI-8, high physical activity with markedly elevated nHDI-8, as well as low physical activity combined with elevated pHDI-8. Thus, the RMD approach identified individuals characterized by atypical joint configurations of physical activity and dietary behaviors rather than exclusively by extreme values on any single dimension.

**Figure 2:**
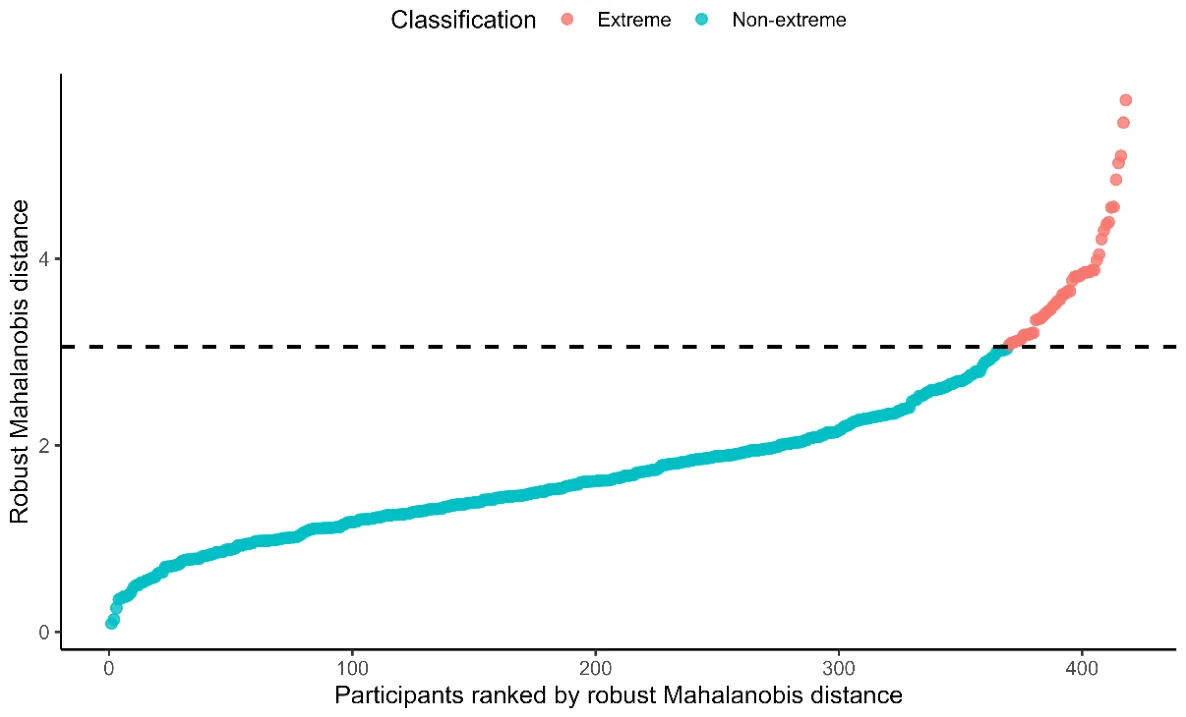
Ranked distribution of robust Mahalanobis distances (RMD) across participants. The dashed horizontal line represents the predefined cutoff for identifying extreme multivariate physical activity–dietary behavioral profiles (RMD > 3.06). Participants exceeding the cutoff were classified as extreme.

**Figure 3:**
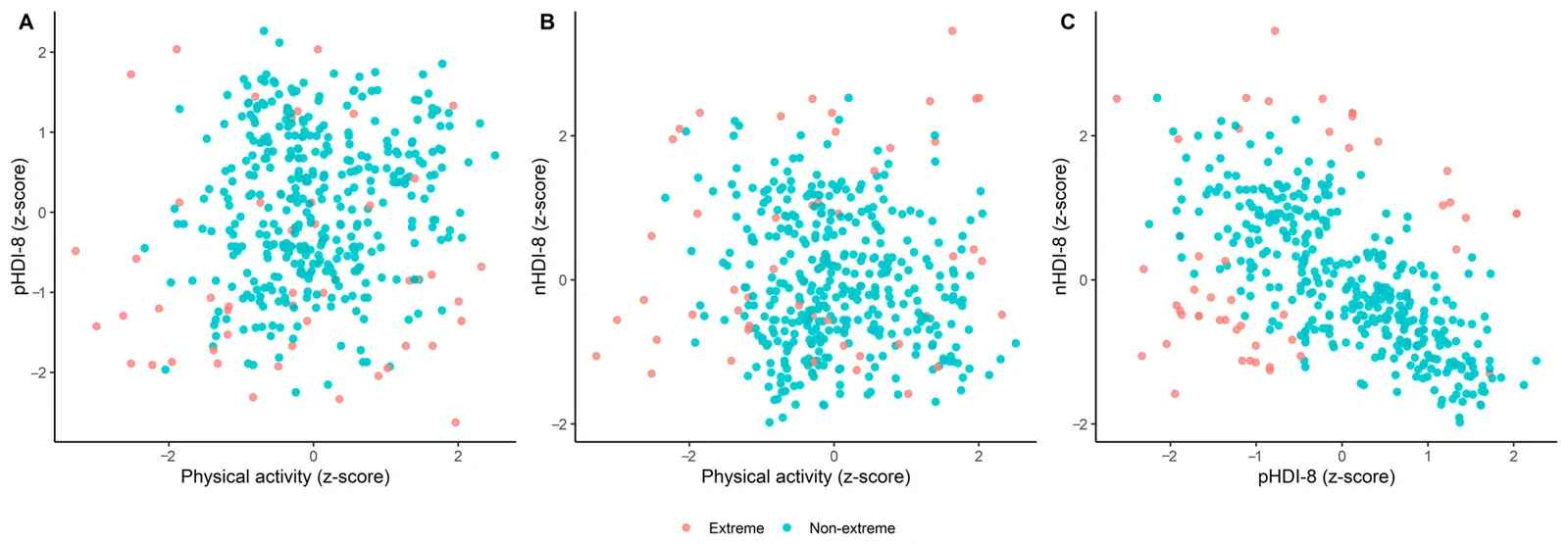
Pairwise projections of extreme and non-extreme physical activity–dietary behavioral profiles. Standardized behavioral dimensions are shown for (**A**) physical activity (PA) and pHDI-8, (**B**) PA and nHDI-8, and (**C**) pHDI-8 and nHDI-8. Extreme profiles were identified using robust Mahalanobis distance (RMD > 3.06).

The ranked distribution of RMD values showed a distinct upper tail beyond the predefined multivariate cutoff (Figure 2). Pairwise projections of the standardized behavioral space further demonstrated that extreme observations occurred across different regions of the PA–dietary behavior space, indicating heterogeneous configurations underlying multivariate extremeness (Figure 3).

### 3.3. Bayesian Identification of Physical Activity–Dietary Behavioral Profiles—Mixture/Latent Classes

Bayesian finite-mixture modelling indicated that a two-profile solution provided the most stable among the evaluated models, converging on a representation of the joint physical activity–dietary behavior space. The K = 2 model showed satisfactory convergence and sampling diagnostics, whereas models with three or more components exhibited substantial between-chain instability and poor convergence (Tables S1 and S2). The three-component model remained unstable following targeted refitting with stricter sampling settings (Table S2). The two-profile model also showed substantially better out-of-sample predictive performance than the single-profile model (ELPD difference = 50.3, SE = 10.3; LOOIC: 3317.1 vs. 3417.6) (Table S3). PSIS-LOO diagnostics were satisfactory for both models, with all Pareto k values < 0.50. Accordingly, the two-profile solution was retained as an exploratory representation of behavioral heterogeneity rather than evidence of a definitive two-profile population structure.

Detailed posterior diagnostics confirmed satisfactory sampling performance for the two-component model (maximum R-hat = 1.003, minimum ESS = 1517, no divergent transitions, and no maximum-tree depth exceedances; Tables S1 and S6). Posterior predictive checks indicated generally adequate model fit, with the observed distributions of PA, pHDI-8, and nHDI-8 broadly reproduced by posterior predictive simulations (Figure S1). Observed means, standard deviations, and pairwise correlations fell within the corresponding 95% posterior predictive intervals, with posterior predictive *p*-values ranging from 0.478 to 0.570 (Table S7).

**Figure 4:**
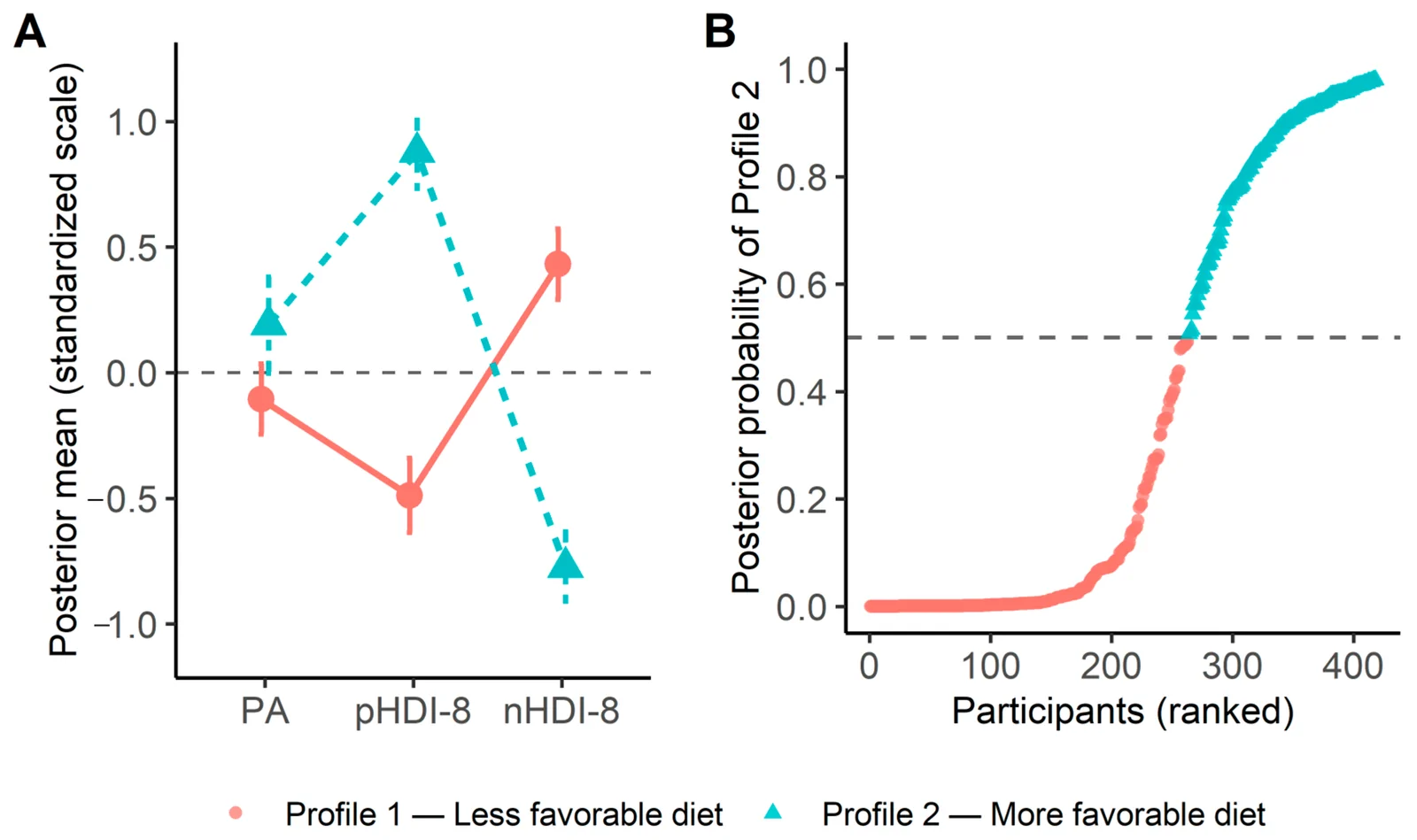
Bayesian identification and posterior classification of physical activity–dietary behavioral profiles. (**A**) Posterior means and 95% credible intervals for standardized physical activity (PA), pro-Healthy Diet Index (pHDI-8), and non-Healthy Diet Index (nHDI-8) in the two-profile Bayesian mixture solution. The horizontal dashed line in panel A denotes the sample mean (z = 0). (**B**) Individual posterior probabilities of membership in Profile 2, ordered from lowest to highest. Points indicate participants assigned to Profile 1 (less favorable dietary profile) or Profile 2 (more favorable dietary profile) according to their highest posterior membership probability. The horizontal dashed line at 0.50 represents the threshold for modal profile assignment.

The two profiles differed predominantly in dietary behaviors (Table 3). Profile 1 comprised 263 participants (62.9%) and was characterized by lower pHDI-8 (posterior mean = −0.49, 95% CrI: −0.64 to −0.34) and higher nHDI-8 (0.43, 95% CrI: 0.29 to 0.57). Physical activity was slightly below the sample mean (−0.11, 95% CrI: −0.24 to 0.04). Profile 2 included 155 participants (37.1%) and showed the opposite dietary configuration, with higher pHDI-8 (0.88, 95% CrI: 0.74 to 1.01) and lower nHDI-8 (−0.77, 95% CrI: −0.91 to −0.63). Physical activity was slightly above the sample mean (0.19, 95% CrI: −0.00 to 0.38) (Figure 4A). Thus, separation between the profiles was primarily attributable to dietary behavior, whereas differences in physical activity were comparatively modest.

Posterior classification was generally well defined. The mean maximum posterior membership probability was 0.90 (median = 0.96), with 80.1% of participants having a probability ≥0.80 and 67.2% ≥0.90. Classification certainty was higher for Profile 1 (mean = 0.93; median = 0.99) than for Profile 2 (mean = 0.85; median = 0.90) (Figure 4B).

### 3.4. Sex Differences in Extreme and Discordant Behavioral Profiles

Bayesian profile membership differed substantially by sex (χ^2^(1) = 61.86, *p* < 0.001). Profile 2, characterized by more favorable dietary behaviors, included 54.8% of women compared with 17.6% of men. Accordingly, women had higher odds of membership in Profile 2 than men (OR = 5.68, 95% CI: 3.62–8.92; Table 4).

Discordant PA–dietary configurations were observed in 49.5% of the total sample and were more frequent among women than men (57.1% vs. 41.2%; χ^2^(1) = 10.51, *p* = 0.001), corresponding to an OR of 1.90 (95% CI: 1.29–2.80). However, the type of discordance differed markedly by sex (χ^2^(3) = 133.08, *p* < 0.001). The lower-PA/more-favorable-diet configuration was observed in 50.2% of women but only 5.5% of men, whereas the higher-PA/less-favorable-diet configuration occurred in 6.8% and 35.7%, respectively. In the multinomial model, relative to the concordant favorable configuration, women had higher odds of the lower-PA/more-favorable-diet configuration (OR = 7.24, 95% CI: 3.47–15.12), but lower odds of the higher-PA/less-favorable-diet configuration (OR = 0.15, 95% CI: 0.08–0.30; Table 4).

A sensitivity analysis using sex-specific cutoffs showed that discordant PA–dietary configurations remained more frequent among women than men (47.9% vs. 34.2%; χ^2^(1) = 8.15, *p* = 0.004; Table S4). Sex differences across the four behavioral configurations also remained statistically significant (χ^2^(3) = 11.36, *p* = 0.010), although the between-sex differences in their distribution were markedly attenuated compared with the pooled-sample classification (χ^2^(3) = 133.08, *p* < 0.001). Overall, 70.3% of participants retained their original four-category classification, indicating that the distribution of specific behavioral configurations was sensitive to the choice of reference cutoffs.

Discordance per se was not associated with multivariate extremeness: 12.1% of participants with discordant configurations and 11.4% of those with concordant configurations were classified as RMD-extreme (OR = 1.07, 95% CI: 0.59–1.94; *p* = 0.823). However, the prevalence of RMD-extreme observations differed across the four specific behavioral configurations (χ^2^(3) = 16.10, *p* = 0.001), indicating that multivariate extremeness depended on the particular PA–dietary configuration rather than on discordance alone.

**Table 4:** Sex differences in Bayesian profiles and concordant and discordant physical activity–dietary behavioral configurations.

Outcome/Configuration	Women, *n* (%)	Men, *n* (%)	OR (Women vs. Men)	95% CI	*p*
Bayesian Profile 2	120 (54.8)	35 (17.6)	5.68	3.62–8.92	<0.001
Any discordance	125 (57.1)	82 (41.2)	1.90	1.29–2.80	0.001
Concordant favorable	58 (26.5)	42 (21.1)	Reference	—	—
Concordant unfavorable	36 (16.4)	75 (37.7)	0.35	0.20–0.61	<0.001
Discordant: higher PA/less favorable diet	15 (6.8)	71 (35.7)	0.15	0.08–0.30	<0.001
Discordant: lower PA/more favorable diet	110 (50.2)	11 (5.5)	7.24	3.47–15.12	<0.001

ORs represent the odds for women relative to men. For the multinomial model, concordant favorable was the reference behavioral configuration. PA, physical activity; CI, confidence interval.

## 4. Discussion

The present study demonstrates substantial individual heterogeneity in the joint distribution of physical activity and dietary behaviors that is not apparent from average between-sex comparisons. Robust multivariate analysis identified 11.7% of participants as exhibiting extreme PA–dietary behavioral profiles, with a significantly higher prevalence among men than women (15.1% vs. 8.7%; *p* = 0.042). Bayesian mixture modelling yielded a converged, exploratory two-profile solution, differentiated predominantly by dietary behaviors rather than physical activity, with the more favorable dietary profile substantially more frequent among women. Importantly, nearly half of the sample exhibited discordant PA–dietary configurations, and both their prevalence and direction differed markedly by sex: women more frequently combined lower PA with more favorable dietary behaviors, whereas men more frequently combined higher PA with less favorable dietary behaviors. Discordance itself was not associated with multivariate extremeness, indicating that discordant and extreme profiles represent distinct, complementary dimensions of behavioral heterogeneity.

Bayesian mixture modelling provided a complementary perspective on behavioral heterogeneity, with the exploratory two-profile solution showing satisfactory convergence and posterior predictive fit. The profiles were differentiated predominantly by dietary behaviors: one combined lower pHDI-8 with higher nHDI-8, whereas the other showed the opposite, more favorable dietary configuration. Differences in PA were comparatively modest, indicating that PA was not the principal dimension separating the recurring profiles in this population. Previous person-centered studies similarly indicate that lifestyle behaviors do not necessarily cluster into uniformly healthy or unhealthy patterns. A recent systematic review of studies in young people identified 172 behavioral clusters, of which 98 represented mixed combinations of diet, physical activity, and sedentary behavior [1]. This heterogeneity is illustrated by the HELENA study, in which only 18% of European adolescents exhibited consistently healthy and 21% consistently unhealthy patterns across physical activity, sedentary behavior, and diet, whereas the remaining participants were characterized by mixed behavioral configurations [17]. Similar discordance has also been observed in adults: Patterson et al. identified groups characterized by a good diet but sedentary behavior and, conversely, by relatively poorer diet quality combined with high physical activity [18]. Together, these findings support the view that physical activity and dietary behaviors may combine in distinct and partly discordant configurations within the same population. Conversely, studies reporting a tendency for higher PA to cluster with healthier dietary behaviors would suggest a greater degree of behavioral concordance than observed here [19,20]. The present Bayesian analysis extends this evidence by showing that, when PA, healthy dietary behaviors, and unhealthy dietary behaviors were modelled jointly, the exploratory two-profile representation was differentiated primarily by dietary behaviors rather than by a parallel PA–diet gradient. This interpretation is consistent with previous person-centered studies showing that physical activity and dietary behaviors may vary relatively independently within behavioral profiles. In a previous study of Polish late adolescents, only partial congruence between physical activity and dietary behavior patterns was observed using a cophylogenetic clustering approach [21]. Similarly, Iannotti and Wang (2013) [22] identified both a profile combining high PA with healthier eating and another characterized by low PA together with low consumption of both healthy and unhealthy foods, whereas Graf and Cecchini (2018) [8] reported that sufficient PA frequently coexisted with average or unhealthy dietary patterns. The substantially greater representation of women in the more favorable dietary profile is also consistent with studies reporting generally healthier food choices or higher diet quality among women [23,24].

The clearest evidence of individual behavioral heterogeneity emerged from the analysis of concordant and discordant configurations. Nearly half of the participants exhibited discordance between PA and dietary favorability, demonstrating that higher activity did not necessarily coexist with more favorable dietary behaviors and that lower activity did not necessarily accompany a less favorable diet. This finding is consistent with previous evidence showing weak or inconsistent relationships between PA and dietary behaviors. In a longitudinal study of adults, within-person changes in PA and fruit and vegetable intake were essentially unrelated, despite a modest between-person association [9], while objectively measured activity and sedentary behavior were not significantly associated with the dietary measures examined in older adults [25]. Similarly, changes in PA during an intervention among sedentary women did not predict corresponding improvements in eating behaviors [26]. However, this relationship is not uniform across populations, as positive associations between PA and diet quality or favorable dietary patterns have also been reported [4,15]. Importantly, person-centered studies provide more direct evidence that these behaviors may coexist in discordant configurations: in European adolescents, profiles combining high PA with a lower-quality diet as well as low PA with a higher-quality diet were identified [17]. Thus, associations observed at the group level may conceal substantial heterogeneity in how physical activity and dietary behaviors are combined within individuals. Other studies, however, have reported positive clustering of health-promoting behaviors, with more physically active individuals also tending to report healthier dietary patterns [27,28]. The present findings suggest that such average associations may conceal substantial within-population heterogeneity. Moreover, discordance was strongly sex-specific in its direction. Approximately half of the women combined lower PA with more favorable dietary behaviors, whereas more than one-third of the men combined higher PA with less favorable dietary behaviors. However, sensitivity analyses using sex-specific cutoffs showed that these directional differences were partly dependent on the pooled-sample reference values, although the overall association between sex and behavioral discordance remained statistically significant. This pattern is broadly consistent with evidence that men tend to report higher levels of PA and are more likely to meet PA recommendations than women [29], whereas women have more frequently been characterized by healthier food choices, including greater consumption of fruit, vegetables, and other health-promoting foods and lower consumption of some less favorable food groups [23,24,30]. However, sex differences in diet are not uniform across dietary components or populations and should therefore not be interpreted as indicating consistently higher overall diet quality among women. Importantly, the present analysis extends these separate observations by demonstrating that sex differences in PA and dietary behaviors can coexist within individuals as contrasting PA–dietary configurations. Finally, discordance was not associated with RMD-defined extremeness, indicating that behavioral mismatch and multivariate atypicality represent distinct dimensions of behavioral heterogeneity.

From a practical perspective, these findings argue against classifying individuals globally as having a “healthy” or “unhealthy” lifestyle on the basis of a single behavior. Both sufficient physical activity and healthy dietary behaviors are independently recognized as major components of the prevention of noncommunicable diseases [31,32], yet a favorable status in one behavioral domain does not necessarily imply a similarly favorable pattern in another [33,34]. The present findings provide an individual-level illustration of this problem: highly active individuals may still exhibit relatively unfavorable dietary behaviors, whereas individuals with favorable dietary patterns may have comparatively low PA. This distinction may be relevant because different lifestyle components can contribute independently to health and functional status [35,36,37]. For example, previous research in older Polish women demonstrated differences in structural and functional health markers according to habitual lifestyle and physical activity [38]. Together, these observations reinforce the value of moving beyond global lifestyle labels toward multidimensional, person-centered assessment. Joint consideration of PA and dietary behaviors may help identify which component of an individual’s behavioral profile represents the more relevant target for intervention [39]. The pronounced sex differences in the direction of discordance observed in the present study may additionally inform targeted health-promotion strategies, although sex should not substitute for individual behavioral assessment [40]. Whether the behavioral profiles identified here also predict differential responses to lifestyle interventions remains to be established [41,42]. Evidence regarding sex-specific responses to lifestyle interventions remains limited and inconsistent: systematic reviews suggest that sex/gender may influence intervention effectiveness in some settings, but available findings do not support a uniform differential response between women and men [43,44]. Nevertheless, such a possibility is plausible given the substantial inter-individual variability in response to exercise and multicomponent interventions. Previous studies have identified responders and non-responders across body-composition, cardiovascular, and fitness outcomes and have shown that baseline characteristics, including body composition, sex, and biological maturation, may contribute to differences in responsiveness [45,46].

The identified behavioral profiles should also be considered within a broader social and environmental context. Socioeconomic circumstances, cultural norms, access to healthy foods, and opportunities for physical activity may influence both dietary choices and activity patterns. Consequently, differences in PA–dietary configurations may reflect not only individual behavioral preferences but also differences in available resources and environmental conditions. As these contextual factors were not examined in the present analyses, their potential contribution to profile membership and behavioral discordance remains uncertain. In university populations, academic background may represent an additional contextual factor, as differences in health-related education could influence knowledge, physical activity, and dietary behaviors.

Overall, the findings demonstrate that behavioral heterogeneity extends beyond average differences in physical activity and dietary behaviors. Robust multivariate atypicality, Bayesian-derived behavioral profiles, and PA–diet discordance captured complementary aspects of this heterogeneity, with the latter showing sex-related differences that were partly sensitive to the choice of classification cutoffs. Together, these findings support a multidimensional, person-centered perspective in which physical activity and dietary behaviors should be considered jointly rather than interpreted as interchangeable indicators of an overall healthy or unhealthy lifestyle.

The strengths of this study include the use of complementary analytical approaches addressing different aspects of behavioral heterogeneity. Robust Mahalanobis distance identified atypical multivariate configurations without relying on single-variable thresholds, while Bayesian mixture modelling provided probabilistic identification of recurring profiles and quantified uncertainty in profile membership. Analysis of concordant and discordant configurations further allowed the direction of PA–dietary mismatch to be examined at the individual level. Several limitations should also be acknowledged. The cross-sectional design precludes causal inference and does not establish whether the identified configurations are stable over time. PA and dietary behaviors were assessed using self-report questionnaires and may therefore be affected by recall and reporting biases. The sample consisted of university students from a physical-activity-oriented academic environment, where physical activity is an integral part of the educational setting. Although no minimum PA threshold was applied as an eligibility criterion, this recruitment context may have resulted in higher habitual PA levels than those observed in the general university student population, potentially influencing the distribution of PA–dietary behavioral configurations and limiting the generalizability of the findings. Furthermore, socioeconomic, cultural, and environmental factors, as well as participants’ specific fields of study, were not recorded or included in the analyses. Consequently, their potential associations with behavioral profile membership and PA–dietary discordance could not be evaluated. In addition, concordance and discordance were defined relative to the standardized distribution within the study sample and should not be interpreted as clinical categories. Although the main findings regarding sex differences in discordance persisted when sex-specific cutoffs were applied, the distribution of individual behavioral configurations was sensitive to the choice of reference cutoffs. Although the two-component model demonstrated satisfactory convergence, predictive performance, and posterior predictive fit, the failure of more complex mixture models to converge precludes conclusions about the definitive number of behavioral profiles. The identified profiles should therefore be interpreted as exploratory summaries of behavioral heterogeneity rather than as established latent subpopulations.

## 5. Conclusions

This study demonstrates that examining physical activity and dietary behaviors jointly at the individual level reveals substantial heterogeneity that is not captured by average between-sex comparisons. Robust multivariate analysis identified a subgroup with extreme PA–dietary configurations, while Bayesian mixture modelling supported two stable profiles differentiated primarily by dietary behaviors rather than physical activity. Importantly, discordant PA–dietary configurations were common, occurring in nearly half of the sample, and showed pronounced sex-specific patterns: women more frequently combined lower physical activity with more favorable dietary behaviors, whereas men more frequently combined higher physical activity with less favorable dietary behaviors. Discordance was not, however, synonymous with multivariate extremeness, indicating that these approaches capture distinct and complementary aspects of behavioral heterogeneity. These findings support moving beyond isolated behaviors and population averages toward joint, person-centered assessment of physical activity and diet, which may provide a more informative basis for identifying behavioral patterns relevant to targeted health-promotion strategies.

## Figures and Tables

**Table 1:** Anthropometric characteristics, body composition, basal metabolic rate, physical activity, and dietary behavior indices in male and female participants.

	Sex = Males, N = 199				Sex = Females, N = 219					
Variables	Mean	95%CI		SD	Mean	95%CI		SD	t	*p*
		Lower	Upper			Lower	Upper			
Body height [cm]	182.19	181.20	183.18	7.10	168.17	167.37	168.97	6.01	21.85	**<0.001**
Body weight [kg]	79.63	78.25	81.00	9.87	60.86	59.65	62.06	9.04	20.29	**<0.001**
BMI [kg/m^2^]	23.97	23.62	24.32	2.47	21.49	21.12	21.85	2.71	9.74	**<0.001**
Body fat mass [kg]	12.99	12.36	13.63	4.60	14.50	13.76	15.24	5.55	−3.02	**0.003**
Body fat percentage [%]	16.17	15.53	16.82	4.61	23.21	22.46	23.96	5.65	−13.86	**<0.001**
Free fat mass [kg]	66.29	65.26	67.32	7.38	46.31	45.68	46.95	4.77	33.14	**<0.001**
IPAQ [MET/minutes/week]	3608.2	3418.5	3797.9	1356.9	3019.8	2886.5	3153.1	1000.9	5.08	**<0.001**
pHDI [scores]	1.92	1.82	2.01	0.66	2.53	2.44	2.62	0.70	−9.19	**<0.001**
nHDI [scores]	1.88	1.79	1.97	0.64	1.18	1.11	1.24	0.50	12.58	**<0.001**

BMI—Body Mass Index, IPAQ—International Physical Activity Questionnaire, pHDI—pro-Healthy Dietary Index, nHDI—non-Healthy Dietary Index, CI—Confidence Interval, SD—Standard Deviation. Physical activity (PA) is presented on its original scale (MET-min/week), whereas pHDI and nHDI are presented as Yeo–Johnson-transformed scores. All three behavioral variables were subsequently standardized to z-scores for multivariate analyses. Significance < 0.05 is presented in bold font.

**Table 2:** Robust Mahalanobis distance-based identification of extreme physical activity–dietary behavioral profiles in the total sample and by sex.

Group	N	Extreme, *n* (%)	Non-Extreme, *n* (%)	RMD Median	RMD Max
Total	418	49 (11.7)	369 (88.3)	1.65	5.70
Women	219	19 (8.7)	200 (91.3)	1.53	5.46
Men	199	30 (15.1)	169 (84.9)	1.79	5.70

Pearson’s χ^2^(1) = 4.13, *p* = 0.042 (without continuity correction). OR = 1.87, 95% CI: 1.02–3.44 (Wald method; men vs. women).

**Table 3:** Bayesian characteristics of the two physical activity–dietary behavioral profiles. PA, pHDI-8, and nHDI-8 are presented on standardized Yeo–Johnson-transformed scales. CrI, Bayesian credible interval.

Characteristic	Profile 1: Less Favorable Dietary Profile	Profile 2: More Favorable Dietary Profile
Assigned participants, *n* (%)	263 (62.9)	155 (37.1)
Estimated profile proportion, θ	0.642	0.358
PA, posterior mean (95% CrI)	−0.106 (−0.244, 0.035)	0.191 (−0.001, 0.381)
pHDI-8, posterior mean (95% CrI)	−0.489 (−0.635, −0.341)	0.878 (0.735, 1.005)
nHDI-8, posterior mean (95% CrI)	0.431 (0.292, 0.573)	−0.774 (−0.909, −0.634)
Maximum posterior probability, mean	0.929	0.845
Maximum posterior probability, median	0.994	0.896
Membership probability ≥0.80%	85.6	71.0
Membership probability ≥0.90%	78.7	47.7

## Data Availability

The data presented in this study are available from the corresponding author upon reasonable request. The data are not publicly available due to ethical and privacy restrictions.

## Supplementary Materials

The following supporting information can be downloaded at: https://www.mdpi.com/article/10.3390/nu18203379/s1, Table S1. Convergence diagnostics for candidate Bayesian mixture models. Rhat, potential scale reduction factor; ESS, effective sample size. Models were estimated using four Markov chain Monte Carlo chains. Models with K = 3–5 were considered unstable because of substantial between-chain instability and/or sampling pathologies. Table S2. Convergence diagnostics and between-chain stability of the targeted three-profile Bayesian mixture model sensitivity analysis. Table S3. PSIS-LOO comparison of the converged Bayesian mixture models. Table S4. Sensitivity analysis of concordant and discordant physical activity–dietary behavioral configurations using pooled-sample and sex-specific cutoffs. Table S5. Between-sex differences in concordant and discordant physical activity–dietary behavioral configurations using pooled-sample and sex-specific cutoffs. Table S6. Posterior parameter estimates and MCMC diagnostics for the two-component Bayesian mixture model. Table S7. Posterior predictive checks for the two-component Bayesian mixture model. Figure S1. Posterior predictive checks for the two-component Bayesian Gaussian mixture model (K = 2). Observed kernel density distributions (blue lines) of standardized physical activity (PA_z), pro-Healthy Dietary Index (pHDI_z), and non-Healthy Dietary Index (nHDI_z) are compared with replicated distributions (gray lines) generated from 40 posterior predictive draws. The overlap between observed and replicated distributions indicates that the exploratory two-component model broadly reproduces the marginal distributions of the three behavioral variables.
