# Supplementary material for: Beyond Average Sex Differences: A Bayesian Analysis of Extreme and Discordant Physical Activity–Dietary Behavioral Profiles in University Students
Source: Nutrients. 2026 Oct 15;18(20):3379. doi: 10.3390/nu18203379 (PMC13708245; doi:10.3390/nu18203379)
Supplement: Supplementary file 1 [file nutrients-18-03379-s001.zip › nutrients-4629448-supplementary.pdf]

Table S1. Convergence diagnostics for candidate Bayesian mixture models. Rhat, potential scale reduction factor; ESS, effective sample size. Models were estimated using four Markov chain Monte Carlo chains. Models with K = 3–5 were considered unstable because of substantial between-chain instability and/or sampling pathologies.

K	Divergences	Max-treedepth transitions	Max Rhat	Min ESS	Status
1	0	0	1.002	3288	Converged
2	0	0	1.003	1517	Converged
3	0	0	3.868	2	Unstable
4	2	0	1.907	3	Unstable
5	2	2618	6.413	2	Unstable

Table S2. Convergence diagnostics and between-chain stability of the targeted three-profile Bayesian mixture model sensitivity analysis

Parameter	Chain 1	Chain 2	Chain 3	Chain 4	Rhat	ESS
θ1	0.166	0.444	0.394	0.557	1.939	3.0
θ2	0.462	0.364	0.378	0.266	1.374	4.9
θ3	0.372	0.192	0.228	0.177	1.827	3.1
PA1	−0.292	−0.105	−0.152	−0.121	1.171	9.9
PA2	−0.060	0.346	0.309	−0.024	1.328	10.4
PA3	0.188	−0.417	−0.288	0.509	1.487	5.8
pHDI1	−0.773	−0.420	−0.500	−0.467	1.546	3.9
pHDI2	−0.403	−0.013	−0.045	0.451	1.677	3.7
pHDI3	0.847	1.001	0.966	0.913	1.212	7.4
nHDI1	0.896	0.286	0.437	0.382	1.778	3.3
nHDI2	0.291	0.124	0.106	−0.358	1.456	5.0
nHDI3	−0.750	−0.909	−0.874	−0.802	1.228	7.3

Footnote: Values for individual chains represent chain-specific posterior means. The targeted K = 3 model was initialized around the same candidate mode and refitted using stricter sampling parameters (adapt_delta = 0.99, max_treedepth = 15). No divergent transitions or maximum-treedepth transitions occurred; however, substantial between-chain differences, elevated Rhat values, and very low ESS persisted, indicating instability of the three-profile solution.

Table S3. PSIS-LOO comparison of the converged Bayesian mixture models.

Model	ELPD-LOO	SE	pLOO	LOOIC	ΔELPD vs. K=2	SE of ΔELPD	Maximum Pareto *k*
K = 1	−1708.8	27.8	9.6	3417.6	−50.3	10.3	0.247
K = 2	−1658.5	27.7	19.3	3317.1	Reference	—	0.395

Footnote: ELPD-LOO, expected log predictive density estimated using Pareto-smoothed importance sampling leave-one-out cross-validation; pLOO, effective number of parameters; LOOIC, leave-one-out information criterion. Higher ELPD-LOO and lower LOOIC indicate better predictive performance. All Pareto k values were <0.50, indicating reliable PSIS-LOO estimates.

Table S4. Sensitivity analysis of concordant and discordant physical activity–dietary behavioral configurations using pooled-sample and sex-specific cutoffs.

Behavioral configuration	Women: pooled cutoffs, n (%)	Women: sex-specific cutoffs, n (%)	Men: pooled cutoffs, n (%)	Men: sex-specific cutoffs, n (%)
Concordant favorable	58 (26.5)	50 (22.8)	42 (21.1)	56 (28.1)
Concordant unfavorable	36 (16.4)	64 (29.2)	75 (37.7)	75 (37.7)
Discordant: higher PA / less favorable diet	15 (6.8)	50 (22.8)	71 (35.7)	42 (21.1)
Discordant: lower PA / more favorable diet	110 (50.2)	55 (25.1)	11 (5.5)	26 (13.1)
Any discordance	125 (57.1)	105 (47.9)	82 (41.2)	68 (34.2)

Table S5. Between-sex differences in concordant and discordant physical activity–dietary behavioral configurations using pooled-sample and sex-specific cutoffs.

Outcome	Pooled-sample cutoffs	Sex-specific cutoffs
Four behavioral configurations, $\chi^2(3)$	133.08 ($p < 0.001$)	11.36 ($p = 0.010$)
Any discordance, $\chi^2(1)$	10.51 ($p = 0.001$)	8.15 ($p = 0.004$)

Footnote: Between-sex differences were assessed using Pearson's chi-square tests without continuity correction. Degrees of freedom are indicated in parentheses.

Table S6. Posterior parameter estimates and MCMC diagnostics for the two-component Bayesian mixture model.

Parameter	Posterior mean	95% CrI	ESS	R-hat
θ_1	0.642	0.560–0.726	1583	1.001
θ_2	0.358	0.274–0.440	1583	1.001
μ PA_1	−0.106	−0.244–0.035	3510	1.000
μ PA_2	0.191	−0.001–0.381	3898	1.000
μ $pHDI_1$	−0.489	−0.635–−0.341	1517	1.000
μ $pHDI_2$	0.878	0.735–1.005	1793	1.002
μ $nHDI_1$	0.431	0.292–0.573	2137	1.001
μ $nHDI_2$	−0.774	−0.909–−0.634	1830	1.001
σ PA_1	1.022	0.934–1.118	4357	1.000
σ $pHDI_1$	0.864	0.783–0.955	2958	1.000
σ $nHDI_1$	0.944	0.862–1.035	4269	0.999
σ PA_2	0.953	0.842–1.082	4206	1.000
σ $pHDI_2$	0.518	0.430–0.625	2005	1.003
σ $nHDI_2$	0.535	0.450–0.633	2064	1.000

Note: θ, posterior mixture proportion; μ, profile-specific mean; σ, profile-specific standard deviation; PA, physical activity; pHDI, pro-Healthy Dietary Index; nHDI, non-Healthy Dietary Index; CrI, credible interval; ESS, effective sample size; R-hat, potential scale reduction factor. All behavioral variables were Yeo–Johnson-transformed and standardized before modelling.

Table S7. Posterior predictive checks for the two-component Bayesian mixture model.

Statistic	Observed	Replicated mean	95% predictive interval	Bayesian p
PA mean	0.000	0.003	−0.132–0.158	0.533
pHDI mean	0.000	0.003	−0.124–0.143	0.520
nHDI mean	0.000	−0.002	−0.135–0.137	0.478
PA SD	1.000	1.010	0.908–1.115	0.570
pHDI SD	1.000	1.005	0.925–1.095	0.513
nHDI SD	1.000	1.002	0.912–1.094	0.515
PA–pHDI correlation	0.129	0.124	−0.014–0.250	0.510
PA–nHDI correlation	−0.059	−0.060	−0.202–0.067	0.508
pHDI–nHDI correlation	−0.549	−0.540	−0.636–−0.436	0.548

Note: PA, physical activity; pHDI, pro-Healthy Dietary Index; nHDI, non-Healthy Dietary Index; SD, standard deviation. Replicated mean represents the posterior predictive mean of each summary statistic. The 95% predictive interval corresponds to the 2.5th and 97.5th percentiles of its posterior predictive distribution. Posterior predictive p-values represent the proportion of replicated statistics equal to or exceeding the observed statistic.

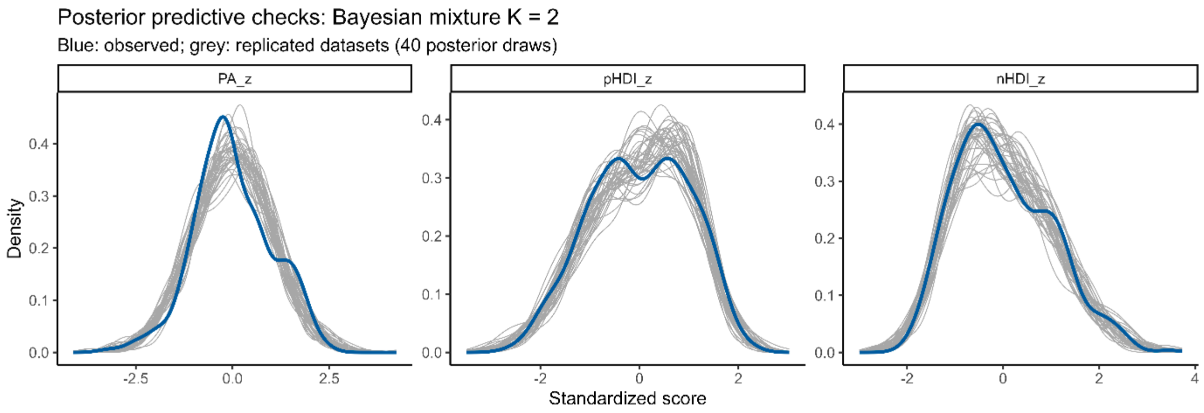

Figure S1. Posterior predictive checks for the two-component Bayesian Gaussian mixture model (K = 2). Observed kernel density distributions (blue lines) of standardized physical activity (PA_z), pro-Healthy Dietary Index (pHDI_z), and non-Healthy Dietary Index (nHDI_z) are compared with replicated distributions (gray lines) generated from 40 posterior predictive draws. The overlap between observed and replicated distributions indicates that the exploratory two-component model broadly reproduces the marginal distributions of the three behavioral variables.
